# Cullin 3^SPOP^ ubiquitin E3 ligase promotes the poly-ubiquitination and degradation of HDAC6

**DOI:** 10.18632/oncotarget.18141

**Published:** 2017-05-24

**Authors:** Yuyong Tan, Yanpeng Ci, Xiangpeng Dai, Fei Wu, Jianping Guo, Deliang Liu, Brian J. North, Jirong Huo, Jinfang Zhang

**Affiliations:** ^1^ Department of Gastroenterology, The Second Xiangya Hospital of Central South University, Changsha 410011, P.R. China; ^2^ Department of Pathology, Beth Israel Deaconess Medical Center, Harvard Medical School, Boston, MA 02215, USA; ^3^ School of Life Science and Technology, Harbin Institute of Technology, Harbin 150001, P.R. China; ^4^ Department of Urology, Huashan Hospital, Fudan University, Shanghai 200040, P.R. China

**Keywords:** HDAC6, SPOP, Cullin 3, ubiquitination, tumorigenesis

## Abstract

The histone deacetylase 6 (HDAC6) plays critical roles in human tumorigenesis and metastasis. As such, HDAC6-selective inhibitors have entered clinical trials for cancer therapy. However, the upstream regulator(s), especially ubiquitin E3 ligase(s), responsible for controlling the protein stability of HDAC6 remains largely undefined. Here, we report that Cullin 3^SPOP^ earmarks HDAC6 for poly-ubiquitination and degradation. We found that the proteasome inhibitor MG132, or the Cullin-based E3 ligases inhibitor MLN4924, but not the autophagosome-lysosome inhibitor bafilomycin A1, stabilized endogenous HDAC6 protein in multiple cancer cell lines. Furthermore, we demonstrated that Cullin 3-based ubiquitin E3 ligase(s) primarily reduced the stability of HDAC6. Importantly, we identified SPOP, an adaptor protein of Cullin 3 family E3 ligases, specifically interacted with HDAC6, and promoted its poly-ubiquitination and subsequent degradation in cells. Notably, cancer-derived *SPOP* mutants disrupted their binding with HDAC6 and thereby failed to promote HDAC6 degradation. More importantly, increased cellular proliferation and migration in *SPOP*-depleted HCT116 colon cancer cells could be partly reversed by additional depletion of *HDAC6*, suggesting that HDAC6 is a key downstream effector for SPOP tumor suppressor function. Together, our data identify the tumor suppressor SPOP as an upstream negative regulator for HDAC6 stability, and *SPOP* loss-of-function mutations might lead to elevated levels of the HDAC6 oncoprotein to facilitate tumorigenesis and metastasis in various human cancers.

## INTRODUCTION

Histone acetyltransferases (HATs) and their counteracting enzymes, histone deacetylases (HDACs) are the Yin and Yang forces that govern tumor initiation, progression, and metastasis largely through the acetylation and deacetylation of histone and non-histone proteins [1]. Currently, 18 potential human HDACs have been reported and grouped into four classes based on their sequence identity, catalytic mechanisms, and homology with yeast histone deacetylases [1, 2]. Specifically, Rpd3-like deacetylases (Class I) consist of four different isoforms: HDAC1, 2, 3, and 8. Hda1-like enzymes (Class II) with six isoforms are further divided into Class IIa (HDAC4, 5, 7, 9) and Class IIb (HDAC6 and 10). Sir2-like deacetylases (Class III) are comprised of seven NAD^+^-dependent sirtuins: Sirt1-7. Finally, HDAC11 is the only known member of the Class IV. As the expression and function of HDACs are frequently dysregulated in multiple types of human cancers, targeting HDACs has become a promising therapeutic option for cancer therapy. To this end, many HDACs inhibitors have been developed for clinical use with four having been approved thus far by the FDA, notably vorinostat, romidepsis, panobinostat and belinostat. However, these pan-HDAC inhibitors which are largely able to inhibit Class I, II and IV enzymes have severe side-effects which limit their prolonged use for cancer therapy. Recently, molecules targeting specific HDAC isoforms have been designed and investigated [3]. Notably, the HDAC6 stands apart from the rest of HDACs because of its unique structure and physiological functions in cells [4, 5].

HDAC6 is a unique member of HDACs due to the fact that it contains two functional catalytic domains and has many non-histone protein targets, such as tubulin [6], HSP90 [7], cortactin [8] and p53 [9]. Hence, HDAC6 is involved in regulating diverse key biological processes including cell motility, cell division, protein trafficking, and apoptosis largely through changing the acetylation status of its target proteins [10, 11]. Moreover, dysregulation of HDAC6 enzymatic activity has been reported to associate with various human diseases including cancers and neurological diseases. Indeed, overexpression of HDAC6 can promote tumorigenesis, invasion, and metastasis [5, 11]. Therefore, these unique features of HDAC6 have made it an interesting potential therapeutic target and HDAC6 specific inhibitors have entered into preclinical and clinical development [5, 10, 11]. Although HDAC6 activity is regulated by posttranslational modifications, such as phosphorylation by Aurora A kinase [12] and extracellular signal-regulated kinase (ERK) [13], or acetylation by p300 [14], the upstream regulator(s), especially ubiquitin E3 ligase(s), regulating the HDAC6 protein stability have not yet been fully elucidated.

The Cullin-Ring ligases (CRLs) are the largest family of E3 ubiquitin ligases and are categorized into seven subfamilies based on Cullin scaffold proteins (Cullin1, 2, 3, 4A, 4B, 5 and 7) [15, 16]. Recently, the CRL3 subfamily of E3 ubiquitin ligases have been linked to various human diseases, such as neurodegeneration and cancer [17]. Like other CRLs family complexes, CRL3 consists of a Cullin scaffold protein (Cullin 3), the RING protein Rbx1, and a variable BTB domain adaptor protein, which serves as a substrate recognition component to recruit protein substrates into the complex for ubiquitination [17]. The Speckle-type POZ (pox virus and zinc finger protein) protein (SPOP) is a CRL3 family adaptor protein, which comprises two conserved functional domains: an N-terminal meprin and TRAF homology (MATH) domain that is primarily involved in substrate recognition and a C-terminal bric-a-brac, tramtrack and broad complex (BTB)/POZ domain that binds Cullin 3 to form a functional multi-component E3 ligase complex (Cullin 3^SPOP^) [18].

In this study, we report that Cullin 3^SPOP^ is a *bona fide* E3 ubiquitin ligase for HDAC6, which functions to earmark HDAC6 for poly-ubiquitination and subsequent degradation. Importantly, cancer-derived SPOP mutations disrupt their binding with HDAC6 and are thereby deficient in promoting HDAC6 destruction. Physiologically, depletion of *SPOP* could increase the cellular proliferation and migration, which could be partly reversed by additional depletion of *HDAC6*. These findings provide the molecular insights into how loss of SPOP function may be mechanistically linked with elevated HDAC6 protein levels that may facilitate tumorigenesis and metastasis in cancer patients with *SPOP*-mutant genetic status.

## RESULTS

### The ubiquitin proteasome system, but not autophagosome-lysosome, controls the protein stability of HDAC6 in cells

**Figure 1:**
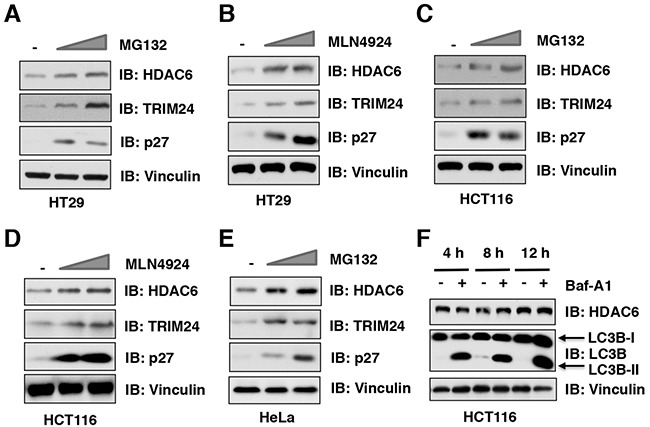
The ubiquitin proteasome system, but not autophagosome-lysosome, controls the protein stability of HDAC6 in cells **(A-B)** Immunoblot (IB) analysis of whole cell lysates (WCLs) derived from HT29 cells treatment with 10 μM, 20 μM MG132 (**A**) or 1 μM, 2 μM MLN4924 (**B**) for 12 hours before harvesting. **(C-D)** IB analysis of WCLs derived from HCT116 cells treatment with 10 μM, 20 μM MG132 (**C**) or 1 μM, 2 μM MLN4924 (D) for 12 hours before harvesting. (**E**) IB analysis of WCLs derived from HeLa cells treatment with 10 μM or 20 μM MG132 before harvesting. (**F**) IB analysis of WCLs derived from HCT116 cells treatment with/without 100 nM Bafilomycin A1 (Baf-A1) at indicated time course before harvesting.

The ubiquitin proteasome system and the autophagosome-lysosome are two major pathways that regulate protein degradation in cells. MG132 is a peptide aldehyde (Z-Leu-Leu-Leu-al) that selectively blocks the proteolytic activity of the 26S proteasome to reduce the degradation of ubiquitin-conjugated proteins in mammalian cells [19]. MLN4924 is a specific inhibitor of the NEDD8-activating enzyme (NAE), which inactivates CRL containing E3 ubiquitin ligases through inhibiting Cullin neddylation [20], thereby stabilizing downstream substrates of various CRLs [21]. Importantly, we found that both MG132 and MLN4924 treatment stabilized HDAC6 at the endogenous level in multiple cancer cell lines including HT29 (Figure 1A-1B and Supplementary Figure 1A), HCT116 (Figure 1C-1D and Supplementary Figure 1B), and HeLa (Figure 1E). In addition to the ubiquitin proteasome system, the autophagosome-lysosome is another important pathway involved in regulating protein degradation in cells (Supplementary Figure 1C). To explore whether HDAC6 stability is also controlled by the autophagosome-lysosome, we utilized the well-characterized bafilomycin A1 inhibitor to block the autophagosome-lysosome pathway [22, 23]. Notably, although bafilomycin A1 treatment could dramatically elevated the protein abundance of LC3B-II, a biomarker of autophagy, it did not lead to significant changes of HDAC6 protein level in various cancer cell lines including HCT116 (Figure 1F), DLD1 (Supplementary Figure 1D), and HeLa cells (Supplementary Figure 1E). Together, these results suggest that the ubiquitin proteasome system, but not autophagosome-lysosome, might control the protein stability of HDAC6 in cells.

### HDAC6 protein stability is negatively regulated by the Cullin 3 family of E3 ubiquitin ligases

**Figure 2:**
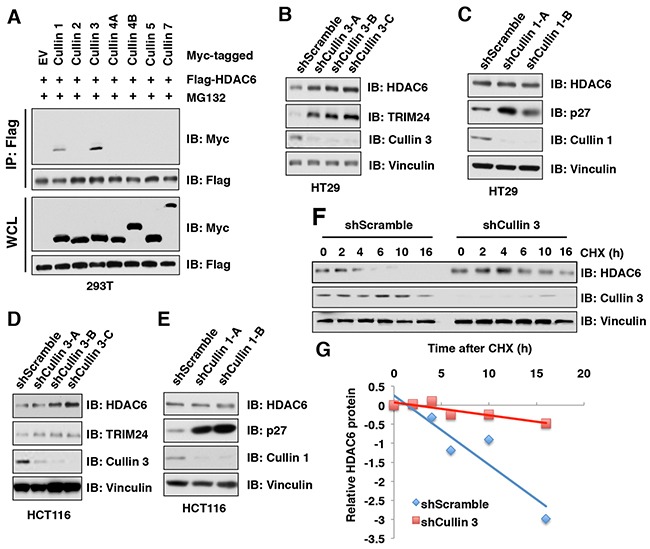
HDAC6 protein stability is negatively regulated by the Cullin 3 family E3 ligase (**A**) IB analysis of WCLs and immunoprecipitates (IP) derived from 293T cells transfected with Flag-HDAC6 and Myc-Cullins constructs as indicated and treated with 10 μM MG 132 before harvesting. **(B-C)** IB analysis of WCLs derived from HT29 cells infected with the indicated lentiviral shCullin 3 (**B**) or shCullin 1 (**C**), respectively. (**D-E**) IB analysis of WCLs derived from HCT116 cells infected with the indicated lentiviral shRNAs against Cullin 3 (**D**) or Cullin 1 (**E**), respectively. **(F-G)** IB analysis of WCLs derived from HT29 cells stably infected with the indicated lentiviral shRNAs and treated with 100 μg/ml cycloheximide (CHX) for indicated times (**F**). Quantification of the band intensities of **(F)** using the ImageJ software (**G**). HDAC6 immunoblot bands were normalized to Vinculin, then normalized to the t = 0 time point.

Our results showed that in addition to MG132, MLN4924 could also stabilize endogenous HDAC6 protein, indicating that the Cullin-Ring family of E3 ligase(s) might be responsible to regulate HDAC6 protein stability. To identify which member of the Cullin-based E3 ubiquitin ligases governs the protein stability of HDAC6, we screened the interaction of Cullin family members with HDAC6 and found that Cullin 1 and Cullin 3, but not the other Cullin family members including Cullin 2, Cullin 4A, Cullin 4B, Cullin 5, and Cullin 7, interacted with HDAC6 in cells (Figure 2A). However, depletion of *Cullin 3*, but not *Cullin 1*, elevated the protein abundance of endogenous HDAC6 in multiple cancer cell lines including HT29 (Figure 2B-2C), HCT116 (Figure 2D-2E), PC3 (Supplementary Figure 2A-2B), and HeLa (Supplementary Figure 2C). These results therefore indicate that only Cullin 3, but not Cullin 1, subfamily of E3 ligases, primarily regulates HDAC6 protein stability in cells. In keeping with this notion, depletion of *Cullin 3* prolonged the half-life of HDAC6 in cells (Figure 2F-2G). These results together reveal that Cullin 3-based E3 ubiquitin ligase(s) plays a crucial role in regulating HDAC6 protein stability in cells.

### SPOP, but not other Cullin 3 family of adaptor proteins, specifically interacts with and promotes HDAC6 poly-ubiquitination and degradation

**Figure 3:**
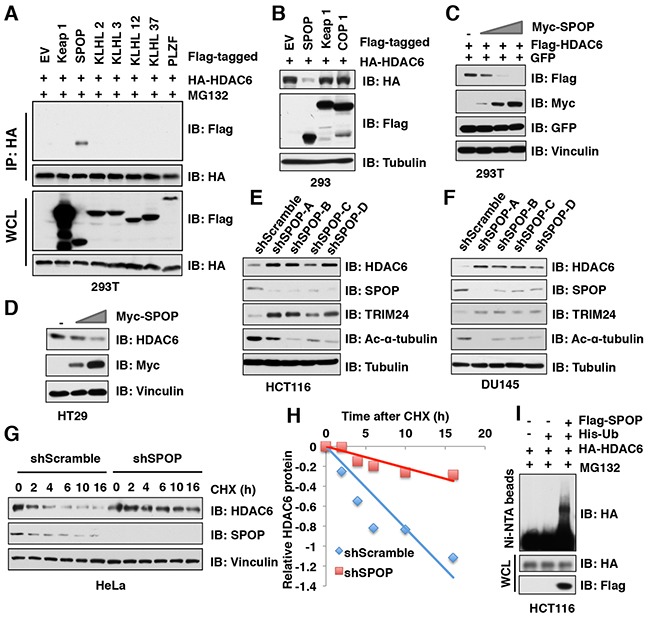
SPOP, but not other Cullin 3 family adaptor proteins, specifically interacts with and promotes HDAC6 poly-ubiquitination and degradation (**A**) IB analysis of WCLs and IP derived from 293T cells transfected with HA-HDAC6 and Flag-tagged Cullin 3 family adaptor constructs as indicated and treated with 10 μM MG132 for 12 hours before harvesting. **(B-C)** IB analysis of WCL derived from 293 (**B**) or 293T (**C**) cells transfected with indicated constructs. (**D**) IB analysis of WCL derived from HT29 cells transfected with indicated constructs. **(E-F)** IB analysis of WCL derived from HCT116 (**E**) or DU145 (**F**) infected with the indicated lentiviral shRNAs against *SPOP* and subjected to puromycin selection for 72 hours before harvesting. **(G-H)** IB analysis of WCL derived from HeLa cells stably infected with the indicated lentiviral shRNAs against *SPOP* and treated with 100 μg/ml CHX for indicated times (**G**). Quantification of the band intensities of (**G**) using the ImageJ software (**H**). HDAC6 immunoblot bands were normalized to Vinculin, then normalized to the t = 0 time point. (**I**) IB of WCL and His pull-down from HCT116 cells transfected with the indicated constructs. Cells were treated with 30 μM MG132 for 6 hours and lysed with denature buffer.

Cullin 3-based E3 ubiquitin ligase(s) utilize one of several adaptor proteins with a BTB/POZ domain to recognize its downstream ubiquitin substrates [17]. To further explore which adaptor protein of Cullin 3-based E3 ubiquitin ligases promotes the poly-ubiquitination and degradation of HDAC6, we examined the interaction of HDAC6 with a panel of BTB domain containing adaptor proteins and found that SPOP, but not other Cullin 3-based E3 ligase adaptor proteins including Keap1, KLHL2, KLHL3, KLHL12, KLHL37, or PLZF interacted with HDAC6 (Figure 3A, Supplementary Figure 3A). Moreover, ectopic expression of SPOP, but not Keap1 nor COP1, dramatically decreased the protein abundance of HDAC6 in cells (Figure 3B). Notably, ectopic expression of SPOP reduced HDAC6 protein levels in a dose-dependent manner at both the exogenous or endogenous levels (Figure 3C-3D). Furthermore, depletion of *SPOP* using several independent shRNAs elevated the endogenous HDAC6 protein abundance in multiple cancer cell lines including HCT116 (Figure 3E), DU145 (Figure 3F), PC3 (Supplementary Figure 3B), and HeLa (Supplementary Figure 3C). In keeping with the notion that SPOP negatively regulates the protein stability of HDAC6, compared with shScramble-treated cells, the half-life of endogenous HDAC6 was prolonged in shSPOP-treated cells (Figure 3G-3H). Consistently, ectopic expression of SPOP could significantly promote the poly-ubiquitination of HDAC6 in cells (Figure 3I). Taken together, these results demonstrate that the Cullin 3^SPOP^ E3 ubiquitin ligase complex is an upstream negative regulator that promotes poly-ubiquitination and subsequent degradation of HDAC6.

### Deletion of the degron motif in HDAC6 confers resistance to SPOP-mediated degradation

**Figure 4:**
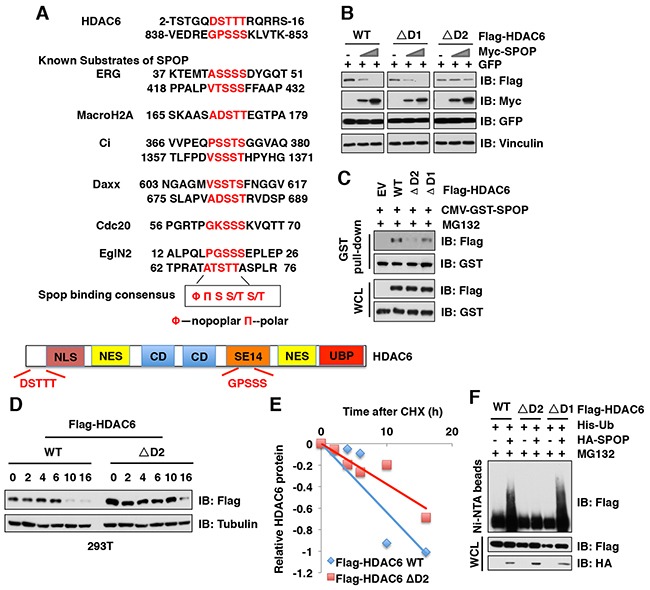
Deletion of the degron motif in HDAC6 confers resistance to SPOP-mediated degradation **(A)** Sequence comparison of HDAC6 with the putative SPOP binding motif (degron) with other known SPOP substrates, and a schematic illustration of HDAC6. NLS: Nuclear localization signal, NES: Nuclear export signal, CD: Catalytic domain, SET14: Cytoplasmic retention domain, UBP: Ubiquitin binding domain. **(B)** IB analysis of WCLs derived from 293T cells transfected with indicated constructs. **(C)** IB analysis of WCL and GST pull-down products derived from 293T cells transfected with indicated constructs. Cells were treated with 10 μM MG132 for 12 hours before harvesting. **(D-E)** IB analysis of WCL derived from 293T cells transfected with indicated Flag-HDAC6 plasmids and treated for indicated times with 100 μg/ml CHX before harvesting (**D**). Quantification of the band intensities of (**D**) using the ImageJ software (**E**). HDAC6 immunoblot bands were normalized to Vinculin, then normalized to the t = 0 time point. **(F)** IB of WCL and His pull-down from HCT116 cells transfected with the indicated constructs. Cells were treated with 30 μM MG132 for 6 hours and lysed with denature buffer.

Previous studies from us and other groups have identified that SPOP substrates including Ci/Gli [24], Daxx [25], MacroH2A [26], ERG [27, 28], AR [29, 30], PTEN [31] SRC-3 [32], DEK [33], TRIM24 [33], Cdc20 [34] and EglN2 [35] share a SPOP-binding motif Φ-Π-S-S/T-S/T (Φ-nonpolar; Π, polar amino acid) (Figure 4A) [18]. Through examining the protein sequence of HDAC6, we found two putative SPOP motifs, or “degrons” in HDAC6: _7_DSTTT_11_ (termed degron 1) and _843_GPSSS_847_ (termed degron 2) (Figure 4A). To identify which degron is required for SPOP-mediated degradation of HDAC6, we generated HDAC6 mutants with deletion of each individual degron and found that HDAC6 harboring deletion of degron 2 (ΔD2), but not degron 1 (ΔD1), became largely resistant to SPOP-mediated HDAC6 degradation (Figure 4B). Consistently, compared to WT or ΔD1 mutant, the HDAC6-ΔD2 mutant lost its ability to bind with SPOP and exhibited an extended half-life in cells (Figure 4C-4E). Furthermore, HDAC6 with ΔD2 mutant, but not the ΔD1 mutant, became resistant to SPOP-mediated poly-ubiquitination in cells (Figure 4F). These results together demonstrate that the degron 2 motif _843_GPSSS_847_ is required for SPOP to recognize and promote the poly-ubiquitination and subsequent degradation of HDAC6 in cells.

### Cancer-associated SPOP mutants fail to interact with and promote the degradation of HDAC6

**Figure 5:**
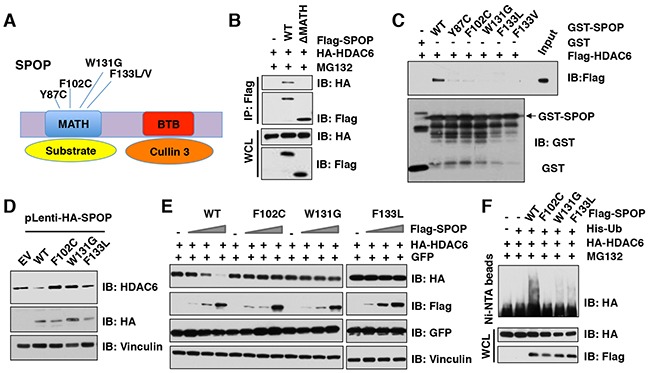
Cancer-associated SPOP mutants fail to interact with and promote the degradation of HDAC6 in cells **(A)** A schematic illustration of SPOP with MATH and BTB domain and cancer-associated mutations. **(B)** Immunoblot (IB) analysis of whole cell lysates (WCL) and immunoprecipitates (IP) derived from HeLa cells transfected with indicated plasmids and treated with 10 μM MG132 before harvesting. **(C)** IB analysis of WCLs and GST pull-down products derived from 293T cells transfected with indicated constructs. **(D)** IB analysis of WCL derived from melanoma WM2664 cells stably expressed with SPOP WT and SPOP mutants. **(E)** IB analysis of WCL derived from 293T cells transfected with indicated constructs. **(F)** IB of WCL and His pull-down from HCT116 cells transfected with the indicated constructs. Cells were treated with 30 μM MG132 for 6 hours and lysed with denature buffer.

Previous studies have shown that *SPOP* is mutated in many human cancers including prostate cancer, endometrial cancer, colorectal cancer, gastric cancer, and thyroid follicular tumors [36–40]. Interestingly, most *SPOP* mutations identified in human prostate cancers, such as Y87C, F102C, W131G, and F133L, are located in the MATH domain of SPOP, which presumably impairs its ability to bind to and recruit substrates into the Cullin 3^SPOP^ complex (Figure 5A). In keeping with this notion, we found that SPOP with the deletion of MATH domain failed to interact with HDAC6 in cells (Figure 5B). To explore whether these cancer-derived SPOP mutants also affects their interaction with HDAC6, we performed GST pull-down assays and found that SPOP mutants including Y87C, F102C, W131G, F133L, or F133V, but not SPOP WT, failed to interact with HDAC6 (Figure 5C). To further determine how these cancer-derived mutations affect endogenous HDAC6 abundance, we generated a melanoma cell line (WM2664) stably expressing SPOP WT or cancer-derived mutants including F102C, W131G, F133L, as well as empty vector (EV) as a negative control. Notably, compared to SPOP WT, cancer-derived SPOP mutants failed to decrease the protein abundance of endogenous HDAC6 in cells (Figure 5D). In keeping with these findings, we further found that ectopic expression of SPOP WT, but not cancer-derived mutants, could promote the degradation of HDAC6 in cells in a dose-dependent manner (Figure 5E), which largely was due to deficiency in promoting the poly-ubiquitination of HDAC6 in cells (Figure 5F). These results demonstrate that cancer-derived mutations of SPOP in the MATH domain are unable to bind and promote the poly-ubiquitination and subsequent degradation of its downstream substrates including the HDAC6 oncoprotein, which might subsequently promote tumorigenesis.

### Depletion of SPOP enhances the cellular proliferation and migration, which can be reversed partly by additional depletion of HDAC6 in colon cancer cells

**Figure 6:**
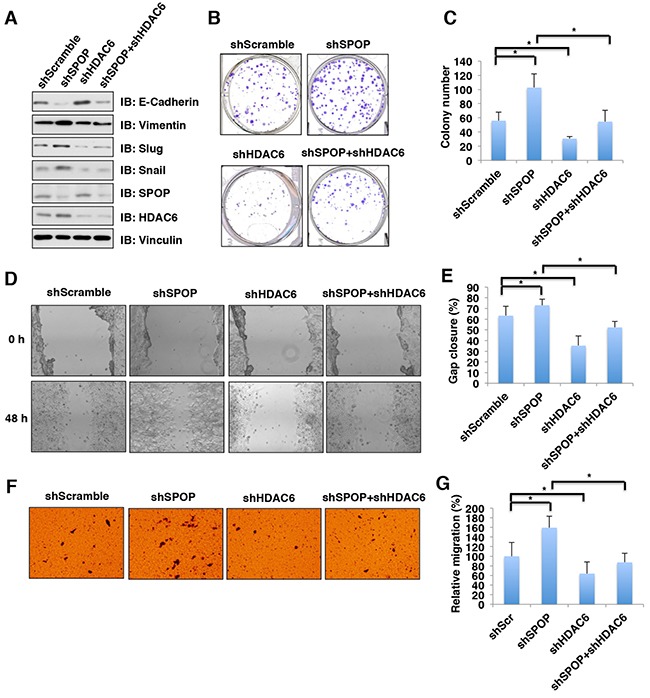
Depletion of SPOP enhances the cellular proliferation and migration, which can be reversed partly by additional depletion of HDAC6 in colon cancer cells **(A)** Immunoblot (IB) analysis of whole cell lysates (WCL) derived from HCT116 infected with the indicated lentiviral shRNAs against SPOP and HDAC6, and subjected to puromycin selection for 72 hours before harvesting. **(B)** Colony formation assay using HCT116 cells described in Figure 6A. **(C)** Quantification of colony number of colony formation assays described in Figure 6B. Data were shown as mean ± SD from three independent experiments. *p < 0.05. **(D)** *In vitro* scratch assay at 0 and 48 hours using HCT116 cells described in Figure 6A. **(E)** Quantification of gap closure of *in vitro* scratch assay described in Figure 6D. Data were shown as mean ± SD from three independent experiments. *p < 0.05. **(F)** Representative images of migrated HCT116 cells described in Figure 6A in migration assays. **(G)** Quantification of migrated cells described in Figure 6F. Data were shown as mean ± SD of three independent experiments. *p < 0.05.

HDAC6 plays an important role in tumorigenesis, invasion and metastasis [5, 11], whereas SPOP has been shown to be a well-characterized tumor suppressor [36–40]. We therefore hypothesized that SPOP exerts its tumor suppressor function partly through regulating the stability of HDAC6 oncoprotein. The epithelial-mesenchymal transition (EMT) is an important process during tumorigenesis and metastasis by which the epithelial cells acquire mesenchymal properties and show reduced intercellular adhesion and increased motility [41]. Previous studies have demonstrated that HDAC6 could induce the EMT in cancer cells [42–45]. To this end, we further explore whether SPOP can regulate the EMT largely through governing the stability of HDAC6. Indeed, we found that depletion of *SPOP* promotes the EMT by decreasing the epithelial marker, E-cadherin and increasing the mesenchymal markers including vimentin, slug and snail (Figure 6A). However, additional depletion of *HDAC6* abrogated the changes in EMT markers observed in cells with depletion of *SPOP* (Figure 6A), suggesting that SPOP regulates the EMT largely through controlling the stability of HDAC6. Furthermore, depleting *SPOP* in HCT116 cells led to elevated cellular proliferation (Figure 6B–6C) and migration (Figure 6D–6G) *in vitro*, which could be largely reversed by additional depletion of *HDAC6* (Figure 6B–6G). These results together support the model that the tumor suppressor SPOP suppresses cellular proliferation and migration largely through negatively regulating the stability of HDAC6 oncoprotein.

## DISCUSSION

In this study, we identified that Cullin 3^SPOP^ is a *bona fide* E3 ubiquitin ligase for HDAC6. Our results showed that MLN4924, an inhibitor for Cullin-based E3 ligase, could stabilize HDAC6 in cells, which indicated that Cullin family E3 ligase might control HDAC6 protein stability (Figure 1). Indeed, HDAC6 could interact with Cullin 1 and Cullin 3, but not other members of Cullin family. However, depletion of *Cullin 3*, but not *Cullin 1*, elevated the protein abundance of HDAC6 at the endogenous level, which suggested that only Cullin 3-based E3 ligase primarily governs the protein stability of HDAC6 in cells (Figure 2). However, additional investigation is warranted to further understand the biological context of the interaction between Cullin 1 and HDAC6 in cells. Furthermore, we identified SPOP as an adaptor protein of Cullin 3 that specifically binds to and promotes the poly-ubiquitinaion and degradation of HDAC6 in a degron-dependent manner (Figures 3 and 4). Although the mRNA levels of HDAC6 upon depletion of *SPOP* were not examined, we performed the half-life assays using cycloheximide to inhibit protein translation and found that depletion of *SPOP* could prolong the half-life of HDAC6 in cells, which indicated that Cullin 3^SPOP^ negatively regulate the stability of HDAC6 largely through the posttranslational modification. Importantly, cancer-derived SPOP mutations disrupted the binding between SPOP and HDAC6 and failed to promote HDAC6 poly-ubiquitination and subsequently degradation (Figure 5), which might lead to elevated levels of HDAC6 to promote tumorigenesis. Functionally, we demonstrated that the tumor suppressor SPOP suppresses the cancer cell proliferation and migration partly through negatively regulating the stability of HDAC6 (Figure 6).

A previous study has reported that the E3 ubiquitin ligase CHIP interacted with HDAC6 and promoted its poly-ubiquitination to suppress abnormal accumulation of the microtubule-binding protein tau, which was correlated with cognitive decline in Alzheimer's disease [46]. Here, our results demonstrated that the ubiquitin E3 ligase Cullin 3^SPOP^ also regulates HDAC6 stability largely through promoting its poly-ubiquitination and subsequent degradation. Hence, it remains unclear how these two different E3 ubiquitin ligases, Cullin 3^SPOP^ and CHIP, respectively, regulate HDAC6 stability. It is plausible that each E3 ubiquitin ligase is working in different cellular contexts, leading to their regulating HDAC6 in the context of cancer (SPOP) or Alzeimer's disease (CHIP) to impact different biological processes that warrants further in-depth investigation.

Given its characterized oncogenic role, HDAC6 has become a promising drug target to treat human cancers, and several HDAC6 specific inhibitors have entered into clinical trials, such as ACY-1215 and ACY-241 [4, 5, 47, 48]. Our results showed that prostate cancer-derived SPOP mutants including Y87C, F102V, W131G, and F133L, which are located in its substrate-recognizing MATH domain, failed to bind to and promote HDAC6 degradation (Figure 5). Moreover, HDAC6 expression levels are elevated in *SPOP*-deficient cells. However, it warrants further in-depth studies to explore whether cancer cells with *SPOP*-mutant genetic status are more sensitive to HDAC6 specific inhibitors due to elevated HDAC6 protein abundance.

Taken together, this study identified the upstream regulator Cullin 3^SPOP^ as a physiological E3 ubiquitin ligase for HDAC6. As *SPOP* is frequently mutated in several cancers, which might lead to a high level of HDAC6 and could alter the sensitivity to HDAC6 specific inhibitors, we envision that our studies may offer a potential novel therapeutic strategy to treat *SPOP*-mutant cancer patients with HDAC6 selective inhibitors.

## MATERIALS AND METHODS

### Cell cultures

293T, 293, 293FT, HCT116, HT29, DLD1, WM2664, and HeLa cells were cultured in DMEM medium (Life Technologies, CA) containing 10% fetal bovine serum (FBS), 100 units of penicillin and 100 mg/ml streptomycin. PC3 and DU145 cells were cultured in RPMI 1640 medium (Corning, NY) containing 10% FBS, 100 units of penicillin and 100 mg/ml streptomycin.

### Plasmids

Myc-Cullin 1, Myc-Cullin 2, Myc-Cullin 3, Myc-Cullin 4A, Myc-Cullin 4B, Myc-Cullin 5, Flag-SPOP WT, Flag-SPOP Y87C, Flag-SPOP F102C, Flag-SPOP W131G, pGEX-4T-1-SPOP, Flag-Keap1, Flag-COP1, shScramble, shCullin 3, shSPOP, and His-ubiquitin constructs were described previously [28]. Myc-Cullin 7 construct was a gift from Dr. James A. DeCaprio (Dana-Farber Cancer Institute). KLHL2 and KLHL3 constructs were kindly offered by Dr. Shinichi Uchida (Tokyo Medical and Dental University). KLHL12 and KLHL37 constructs were purchased from addgene. Flag-SPOP F133L, Flag-SPOP F133V, Flag-PLZF, Flag-HDAC6 and HA-HDAC6 and deletion degron mutants were generated in this study.

### Cell transfection and virus infection

When cells were at 80% confluence, we performed the transiently transfection with indicated constructs using Lipofectamine in Opti-MEM medium (Invitrogen). After 36 hours (h) transfection, cells were harvested and lysed in EBC buffer (50 mM Tris pH 7.5, 120 mM NaCl, 0.5% NP40) supplemented with protease inhibitors (Roche) and phosphatase inhibitors (Calbiochem) for immunoprecipation or immunoblot analysis.

293FT cell line was used for packaging and amplifying lentivirus. The medium containing viruses were collected at 48 hours and 72 hours after transfection. After filtering through 0.45 μM filters, viruses were used to infect cells in the presence of 4 μg/mL polybrene (Sigma-Aldrich). 48 hours post-infection, cells were split and selected using 1 μg/mL puromycin (Sigma-Aldrich) for three days to eliminate the non-infected cells before harvesting.

### Antibodies and reagents

Anti-HDAC6 (7612), mouse monoclonal anti-Myc-Tag (2276), rabbit polyclonal anti-Myc-Tag antibody (2278), anti-Cullin 3 (2759), anti-LC3-B (2775), EMT antibody sampler kit (9782), anti-Ac-α-Tubulin (5335) and anti-GST (2625) antibodies were purchased from Cell Signaling. Anti-TRIM24 (TIF1α, SC-271266), anti-Cullin 1 (SC-11384), anti-HA antibody (SC-805), anti-Tubulin (SC-73242) and anti-p27 (SC-527) antibodies were purchased from Santa Cruz. Anti-SPOP antibody (16750-1-AP) was purchased from Proteintech. Anti-GFP (8371-2) antibody was purchased from Clontech. Polyclonal anti-Flag antibody (F7425), monoclonal anti-Flag antibody (F-3165, clone M2), anti-vinculin antibody (V-4505), peroxidase-conjugated anti-mouse secondary antibody (A-4416), peroxidase-conjugated anti-rabbit secondary antibody (A-4914), anti-HA agarose beads (A-2095) and anti-Flag agarose beads (A-2220) were purchased from Sigma. All antibodies were used in 1:1000 dilutions in 5% non-fat milk for western blot.

MLN4924 was a kind gift from Dr. William Kaelin (Dana-Farber cancer institute). Bafilomycin A1 was kindly provided by Dr. Junying Yuan (Harvard Medical School). MG132 (BML-PI102-0005) was purchased from Enzo life science.

### Immunoprecipitation, GST pull-down assays and western blotting

Cells were harvested and washed once by using cold PBS. The cell pellets were lysed in EBC buffer (50 mM Tris pH 7.5, 120 mM NaCl, 0.5% NP40) supplemented with protease inhibitors (Roche) and phosphatase inhibitors (Calbiochem). The lysates were cleared by centrifugation and the lysates were quantified by Beckman Coulter DU-800 spectrophotometer (Beckman Coulter) using the Bio-Rad protein assay reagent (Bio-Rad Laboratories, CA). 1 mg total lysates were incubated with the appropriate antibody-conjugated beads (2 μg) or bacterial purified GST or GST fused proteins (1 μg) for 4 hours at cold room. Immunocomplexes or GST pull-down products were washed four times with NETN buffer (20 mM Tris, pH 8.0, 100 mM NaCl, 1 mM EDTA, and 0.5% NP-40) and were eluted by boiling for 5 minutes in SDS loading buffer. Bound proteins were resolved by SDS-PAGE and immunoblotted with indicated antibodies.

### Colony formation assays

HCT116 cells with stably expressing shScramble, shSPOP, shHDAC6 or in combination of shSPOP and shHDAC6 were plated in 6-well culture dishes at a density of 1000 cells/well and allowed to growth undisturbed for 10 days. Cells were stained with crystal violet and the colony numbers were counted.

### *In vitro* scratch assay

HCT116 cells were plated in 6-well culture dishes. The cell monolayer was scraped in a straight line with a P200 pipet tip. Photographs of the scratch were taken at 0 h and 48 h. Gap width at 0 h was set to 1. Gap width analysis was performed with ImageJ software. Measurements were taken at multiple defined sites (> 6) along the scratch. Each scratch was given an average of all measurements. Data are expressed as the average of three independent experiments.

### Cell migration assay

5× 10^4^ HCT116 cells was plated in an 8-μm, 24-well plate chamber insert (Corning Life Sciences, catalog no. 3422) with 100 μL serum-free medium at the top of the insert and DMEM medium (Gibco) containing 10 % FBS (500 μL) at the bottom of the insert. Cells were incubated for 24 h and fixed with 4% paraformaldehyde for 15 min. After washing with PBS, cells at the top of the insert were scraped with a cotton swab. Cells adherent to the bottom were stained with 0.5% crystal violet blue for 15 min and then washed with double-distilled H_2_O. The positively stained cells were counted in eight random fields under the microscope, and the average value of eight fields was expressed. All assays were performed in triplicate.

### Statistical analysis

Student *t* tests was used to evaluate significance between groups all other data, and *p*-values indicated. Error bars represent standard deviation. *p < 0.05 indicates significance.

## SUPPLEMENTARY MATERIALS AND FIGURES


