# Supplementary material for: Cullin 3SPOP ubiquitin E3 ligase promotes the poly-ubiquitination and degradation of HDAC6
Source: Oncotarget. 2017 May 24;8(29):47890–901. doi: 10.18632/oncotarget.18141 (PMC5564613; doi:10.18632/oncotarget.18141)
Supplement: Supplementary file 1 [file oncotarget-08-47890-s001.pdf]

Cullin 3[SPOP] ubiquitin E3 ligase promotes the poly-ubiquitination and degradation of HDAC6

SUPPLEMENTARY MATERIALS

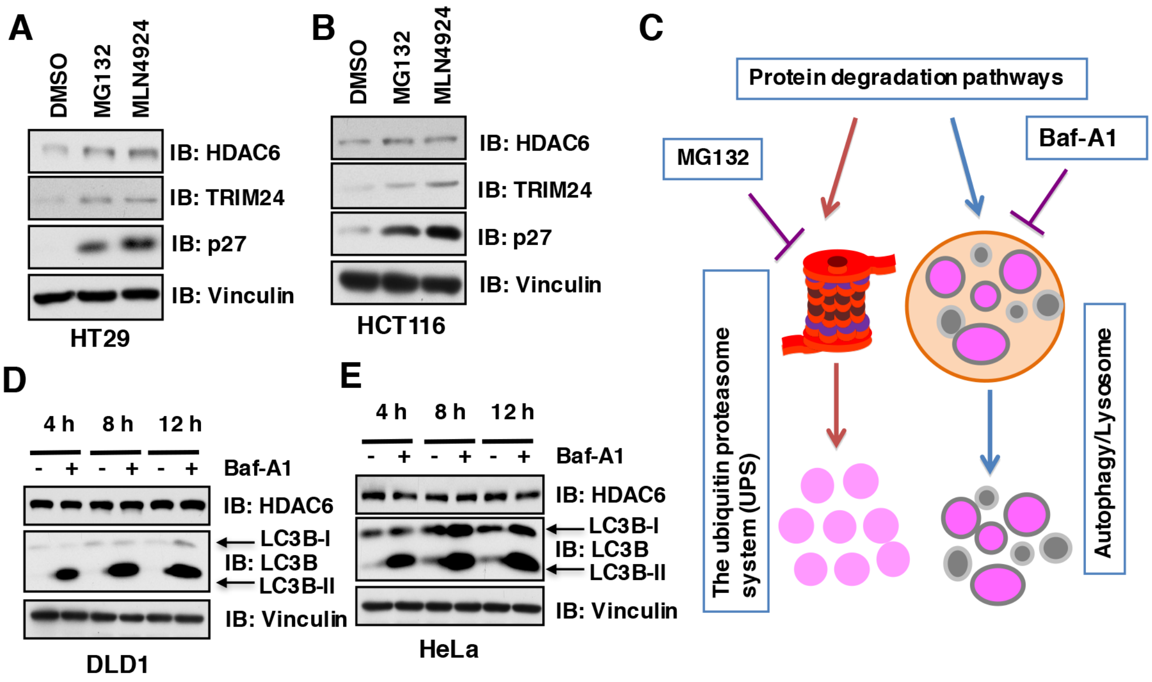

Supplementary Figure 1: The ubiquitin proteasome system, but not autophagosome-lysosome, controls the protein stability of histone deacetylase 6 (HDAC6). **(A-B)** IB analysis of WCL derived from HT29 (**A**) and HCT116 cells (**B**), which were treated with 10 µM MG132 or 1 µM MLN4924 for 12 hours before harvesting. **(C)** A schematic illustration of protein degradation pathways. **(D-E)** IB analysis of WCL derived from DLD1 (**D**) and HeLa cells (**E**) treated with 100 nM Bafilomycin A1 (Baf-A1) for indicated time before harvesting.

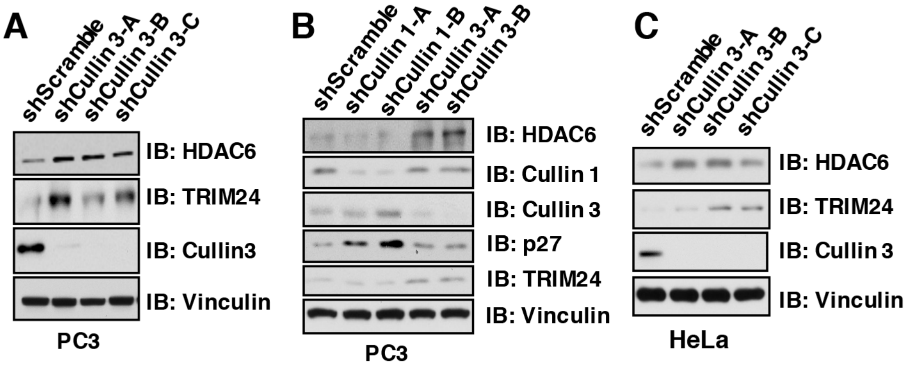

Supplementary Figure 2: Cullin 3-based E3 ligase negatively regulates the protein stability of HDAC6. **(A-B)** IB analysis of WCL derived from PC3 cells infected with the indicated lentiviral shRNAs against Cullin 1 or Cullin 3 as indicated and subjected to 1 µg/ml puromycin selection for 72 hours before harvesting. **(C)** IB analysis of WCL derived from HeLa cells infected with the indicated lentiviral shRNAs against Cullin 3 and subjected to 1 µg/ml puromycin selection for 72 hours before harvesting.

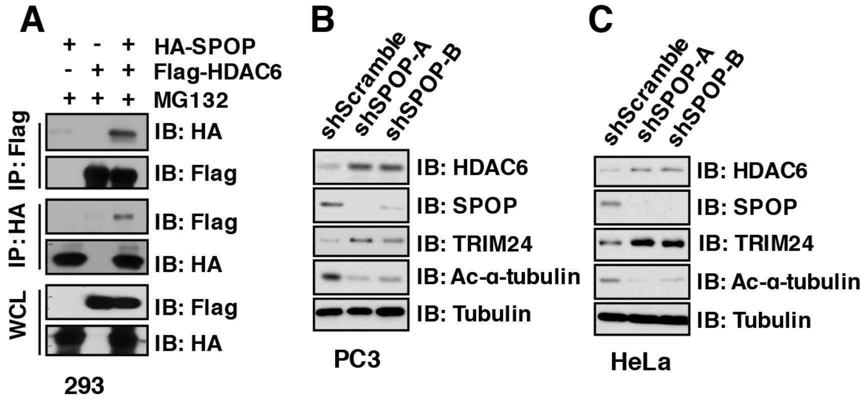

Supplementary Figure 3: Cullin 3^{SPOP} negatively regulates the protein stability of HDAC6. (A) IB analysis of WCL and IP derived from 293 cells transfected with indicated plasmids and treated with 10 µM MG132 before harvesting. **(B-C)** IB analysis of WCL derived from PC3 (**B**) and HeLa cells (**C**) infected with the indicated lentiviral shRNAs against SPOP and subjected to 1 µg/ml puromycin selection for 72 hours before harvesting.
